# SIRL-1 deficiency reveals pro-inflammatory IL-8 axis in inflammatory bowel disease: a novel diagnostic ratio

**DOI:** 10.3389/fimmu.2026.1805306

**Published:** 2026-04-17

**Authors:** Zhengzheng Wang, Shan Li, Qi Tan, Jianmin Sang, Zhen Xu, Xuewei Ding, Xiaoyan Yao, Nianzhen Chen, Shanshan Yu, Ming Zong, Ying Lu, Lan Zhong, Lieying Fan

**Affiliations:** 1 School of Medicine, Tongji University, Shanghai, China; 2 Department of Laboratory Medicine, Shanghai East Hospital, School of Medicine, Tongji University, Shanghai, China; 3 Department of Gastroenterology, Shanghai East Hospital, School of Medicine, Tongji University, Shanghai, China; 4 Research Center for Translational Medicine, Shanghai East Hospital, School of Medicine, Tongji University, Shanghai, China

**Keywords:** biomarker, Crohn’s disease, inflammation, inflammatory bowel disease, interleukin-8, SIRL-1, ulcerative colitis

## Abstract

**Background:**

Inflammatory Bowel Disease (IBD), including Ulcerative Colitis and Crohn’s Disease, is characterized by chronic intestinal inflammation, neutrophil infiltration and elevated pro-inflammatory cytokines like Interleukin-8 (IL-8). While pro-inflammatory mechanisms are well-studied, the contribution of intrinsic inhibitory immune checkpoints to IBD pathogenesis is less understood. This study aimed to assess the expression and function of Signal Inhibitory Receptor on Leukocytes-1 (SIRL-1) in IBD and to evaluate the diagnostic utility of the SIRL-1/IL-8 ratio.

**Methods:**

Peripheral venous blood was collected from 90 participants (IBD patients and healthy volunteers). Neutrophil SIRL-1 protein expression was quantified by flow cytometry, while *VSTM1* (SIRL-1 gene) and IL-8 messenger RNA levels were measured by RT-qPCR. Serum IL-8 concentrations were determined using a flow cytometry microbead array. Statistical analyses included Mann-Whitney U tests, Kruskal-Wallis tests, Spearman correlations, and Receiver Operating Characteristic (ROC) curve analysis.

**Results:**

IBD patients exhibited significant downregulation of SIRL-1 protein and *VSTM1* messenger RNA in peripheral blood neutrophils. This downregulation was constitutive in Crohn’s Disease, irrespective of disease activity. Conversely, serum IL-8 levels were significantly elevated in IBD cohort, driven primarily by the active Ulcerative Colitis subgroup, whereas Crohn’s Disease patients did not show significant elevation. A significant inverse correlation was observed between neutrophil SIRL-1 expression and serum IL-8 (r = -0.5789, *p* = 0.0118). Crucially, the SIRL-1/IL-8 ratio demonstrated superior diagnostic accuracy for distinguishing IBD from healthy controls (AUC = 0.82), outperforming individual markers, and was notably effective in Crohn’s Disease (AUC = 0.86).

**Conclusions:**

Deficiency in SIRL-1 appears to be a permissive factor for the pro-inflammatory IL-8 axis in IBD. The SIRL-1/IL-8 ratio represents a promising novel diagnostic biomarker that presents enhanced precision by reflecting the imbalance between immune regulation and inflammation. Notably, this ratio revealed superior diagnostic value in Crohn’s Disease despite the lack of significant IL-8 elevation, highlighting its utility in capturing intrinsic immune defects.

## 1 Introduction

Inflammatory Bowel Disease (IBD), comprising Ulcerative Colitis (UC) and Crohn’s Disease (CD), is a chronic disorder governed by genetic susceptibility, environmental factors, and immune dysregulation (1–4). A pathological hallmark of IBD is persistent neutrophil infiltration within the intestinal mucosa (5, 6), driving tissue injury via reactive oxygen species (ROS) and pro-inflammatory cytokines (5), particularly Interleukin-8 (IL-8) (7–10). Current research has predominantly focused on pro-inflammatory mediators; however, immune homeostasis relies a balance between activation signals and inhibitory checkpoints. It remains unclear whether IBD inflammation stems solely from excessive activation or involves a failure of intrinsic inhibitory mechanisms.

Signal Inhibitory Receptor on Leukocytes-1 (SIRL-1), encoded by the *VSTM1* gene, is a negative regulator of innate immunity expressed on neutrophils and monocytes (11, 12). SIRL-1 contains intracellular immunoreceptor tyrosine-based inhibitory motifs (ITIMs) that recruit SHP-1/SHP-2 phosphatases (13). Through this mechanism, SIRL-1 dampens downstream signaling pathways triggered by pattern recognition receptors (e.g., TLRs, FcγRs), thereby limiting oxidative burst and NETosis (5, 14–16). Beyond its role in regulating innate immune responses, SIRL-1 has garnered attention as a critical immune checkpoint (17). Studies have shown that genetic variants within the VSTM1 locus can modulate SIRL-1 expression and influence susceptibility to inflammatory conditions, such as atopic dermatitis (18). Furthermore, soluble forms of SIRL-1 (sSIRL-1) have been detected in various inflammatory diseases like rheumatoid arthritis (RA) and respiratory infections, with evidence suggesting that sSIRL-1 levels correlate with disease activity and are released from activated neutrophils (19, 20). While VSTM1-v2, a soluble splice variant of VSTM1, was initially reported to promote Th17 cell differentiation (21), a recent replication study concluded that VSTM1-v2 does not drive human Th17 cell differentiation (22). This highlights the ongoing evolution of understanding around VSTM1 isoforms, but does not diminish the established inhibitory role of membrane-bound SIRL-1 (VSTM1-v1). Moreover, SIRL-1 has been identified to recognize specific ligands, including bacterial phenol-soluble modulins (PSMs), human cathelicidin LL-37 (12), and S100 proteins (23), highlighting its broad role in immune regulation. Despite these advances in understanding SIRL-1’s multifaceted roles in immune regulation, its precise expression and functional significance in the context of IBD remain largely uncharacterized (24). While persistent neutrophil infiltration and elevated IL-8 levels are central to IBD pathogenesis, the contribution of inhibitory immune checkpoints, like SIRL-1, in modulating this inflammatory axis is not fully elucidated in IBD. Bridging this knowledge gap is critical, as an imbalance between inhibitory and activating signals could provide more nuanced insights into disease mechanisms than mere quantification of pro-inflammatory mediators.

We hypothesize that IBD involves a defect in SIRL-1 signaling, where reduced expression weakens the suppression of inflammatory pathways, facilitating unchecked production of chemoattractants such as IL-8 (7, 9). If confirmed, the imbalance between regulation and inflammation could provide superior clinical insight compared to inflammatory markers alone. This study integrates transcriptional, proteomic and serological analysis to characterize the SIRL-1 profile in IBD, defining its relationship with IL-8 and validating the SIRL-1/IL-8 ratio as a diagnostic biomarker.

## 2 Methods

### 2.1 Study population and sample collection

Peripheral venous blood was collected from a total of 90 participants at the Department of Gastroenterology, Shanghai East Hospital. The study cohort comprised 60 patients with confirmed Inflammation Bowel Disease (IBD), including 29 with Ulcerative Colitis (UC) and 31 with Crohn’s Disease (CD), alongside 30 age- and sex-matched healthy volunteers (HC) who served as controls.

Diagnosis of IBD was established via standard clinical, endoscopic, and histological criteria according to the Montreal classification. To assess the impact of disease status, patients were further stratified into active and remission groups based on clinical activity indices or confirmed clinical diagnosis. For UC, disease activity was defined using the modified Mayo Score (active: Mayo > 2; remission: Mayo ≤ 2; mild/moderate/severe activity: scoring 3-5, 6-10, 11-12, respectively). For CD, activity was assessed using the Crohn’s Disease Activity Index (CDAI) (active: CDAI ≥ 150; remission: CDAI < 150; mild/moderate/severe activity: scoring 150-220, 221-450, >450, respectively). The baseline demographic and disease phenotypes characteristics are summarized in **Supplementary Table 1** (https://doi.org/10.6084/m9.figshare.31624945).

### 2.2 Flow cytometric analysis of SIRL-1 expression

A total of IBD patients and HC were involved by flow cytometry. Granulocytes were isolated from fresh peripheral venous blood (processed within 4 hours of collection) via density gradient centrifugation and red blood cell lysis. Cells were washed and blocked with FcR blocking reagent to prevent non-specific binding. Subsequently, cells were stained with fluorochrome-conjugated anti-human SIRL-1 (*VSTM1*) monoclonal antibody (Cat #568957, BD Biosciences). Data acquisition was performed on a BD FACSLyric™ flow cytometer. Granulocytes were identified based on their characteristic forward scatter (FSC) and side scatter (SSC) profiles. Strict gating was applied to the granulocyte population to exclude debris and cells with altered scatter properties indicative of apoptosis or necrosis. Analysis was conducted using FlowJo software, with SIRL-1 expression levels reported as Mean Fluorescence Intensity (MFI) on the gated CD45^+^ neutrophil population.

### 2.3 RNA extraction and quantitative real-time PCR

Total RNA was extracted from peripheral blood granulocytes using the RNAiso Plus Reagent (TaKaRa) according to the manufacturer’s instructions. RNA concentration and purity were assessed using a NanoDrop spectrophotometer (Thermo Scientific). Complementary DNA (cDNA) was synthesized from 1 µg of total RNA using a PrimeScriptTMRT Reagent Kit with gDNA Eraser (TaKaRa). Quantitative PCR was performed using TB Green Premix (TaKaRa) on a Real Time PCR Instrument (Roche Cobas z480). Specific primers for *VSTM1* (F: CCGAGAGCAATGTGACCCTGA; R: GGAGGCTGTTGTCTTGTAGGC), IL8 (CXCL8) (F: TGATTTCTGCAGCTCTGTGTGA; R: GGTCCACTCTCAATCACTCTCA), and the housekeeping gene GAPDH (F: GAACATCATCCCTGCCTCTACTG; R: GGCAGGTTTTTCTAGACGGCAGGT) were utilized. Relative gene expression was calculated using the comparative 2^-△△CT^ method, normalizing target gene expression to the internal control. The samples with insufficient RNA quality or quantity for transcriptional analysis were excluded from amplification.

### 2.4 Flow cytometry microbead array for IL-8 identification

To quantify serum IL-8 levels, a Cytometric Bead Array (CBA) was performed. Quality control (QC) samples containing known concentrations of IL-8 were reconstituted in sample diluent and mixed gently. Serum samples from patients and controls were added to a solution containing capture microbeads specific to IL-8. The fluorescent conjugated antibody was added to the mixture. After incubating in the dark for 2.5 hours, the samples were washed with buffer and centrifuged to remove unbound reagents. The pellets were resuspended in PBS and immediately analyzed via flow cytometry to quantify the IL-8 signal. Concentrations were calculated based on a standard curve generated in the same assay run. Samples processed in the condition of logical constraints were excluded from the further reliable analysis.

### 2.5 Statistical analysis

Data were analyzed using GraphPad Prism (Version 9.5.0). Normality was assessed via Shapiro-Wilk tests. Group comparisons utilized Mann-Whitney U test (for non-normal data). Multiple comparisons used Kruskal-Wallis tests with *post-hoc* corrections. Correlations were evaluated using Spearman’s rank coefficient. Diagnostic performance was assessed via ROC curve analysis (Area Under the Curve [AUC], Sensitivity, Specificity, Youden Index and Diagnostic Odds Ratio [DOR]). *p* < 0.05 was considered significant.

## 3 Results

### 3.1 Study cohort

Detailed demographic analysis confirmed no significant differences in age or gender distribution between IBD subgroups and controls (*p* > 0.05). Based on clinical activity indices, the primary flow cytometry cohort comprised 29 UC patients (13 active, 16 remission) and 31 CD patients (12 active, 19 remission). Due to sample limitations, cohorts varied by assay: Flow cytometry included the full cohort of 60 IBD patients and 30 HC. Transcriptional analysis of *VSTM1* was performed on 56 IBD patients (26 UC, 30 CD) and 31 HC, while serum IL-8 levels were quantified in 37 IBD patients (16 UC, 21 CD) and 36 HC.

### 3.2 Systemic downregulation of SIRL-1 expression

SIRL-1 MFI was significantly reduced in peripheral blood neutrophils of IBD patients compared to controls (*p* = 0.0029). The downregulation were observed in both UC (*p* = 0.0368) and CD (*p* = 0.0285) subgroups. Stratification by clinical activity revealed disease-specific heterogeneity in SIRL-1 expression. In UC, the deficit was specific to active disease (aUC) (*p* = 0.0221 vs. HC), while remission patients were comparable to controls. Conversely, in CD, active patients (aCD) showed no significant deviation, while remission patients (rCD) exhibited a trend toward reduced MFI (*p* = 0.0723).

**Figure 1:**
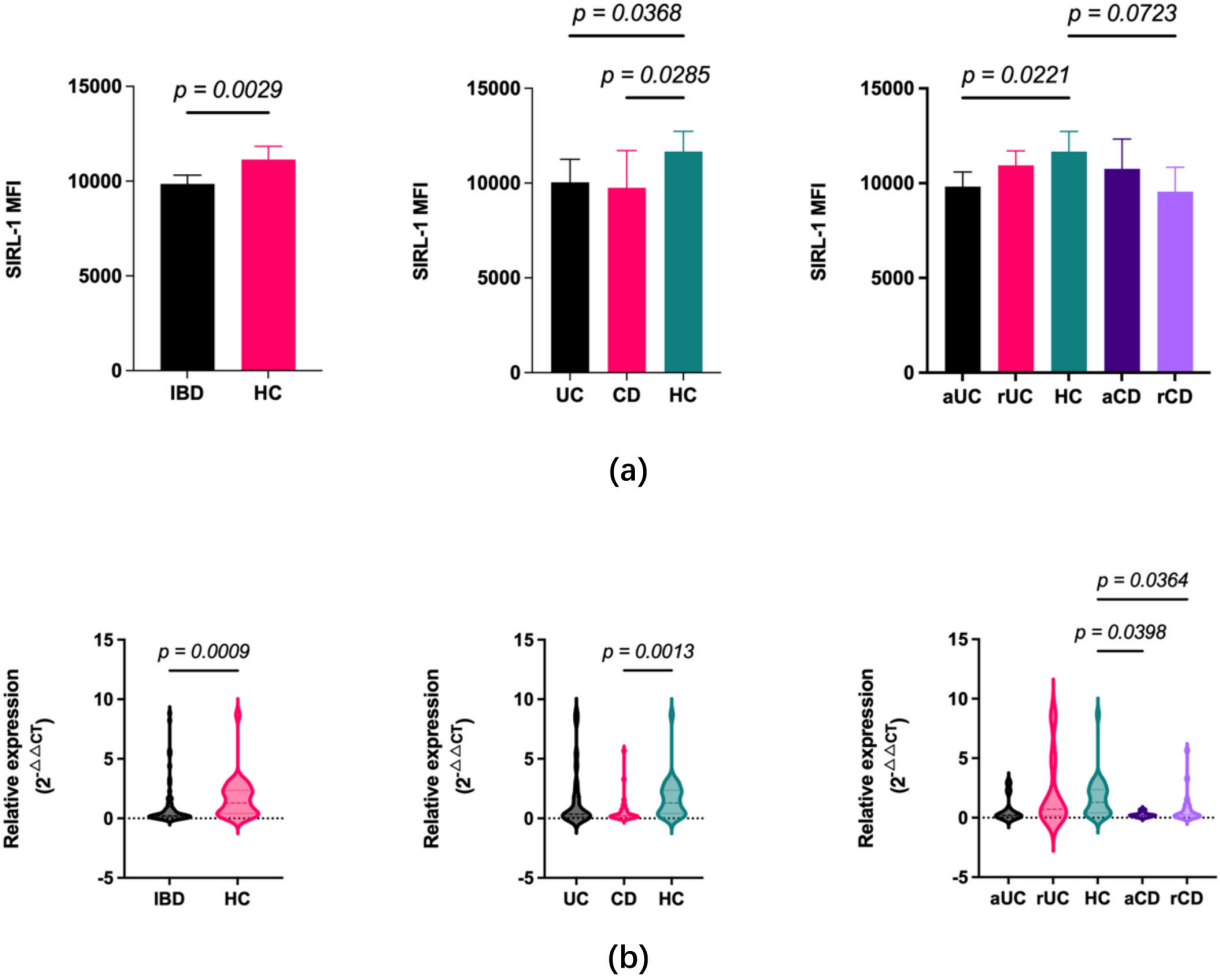
Systemic downregulation of SIRL-1 protein and *VSTM1* mRNA expression in IBD patients. **(a)** Quantification of SIRL-1 surface expression (Mean Fluorescence Intensity, MFI) on peripheral blood neutrophils. Bar graphs illustrate reduced MFI in the total IBD cohort compared to HC, healthy controls (left), with significant reductions observed in both Ulcerative Colitis (UC) and Crohn’s Disease (CD) subgroups (middle). Further stratification by disease activity (right) indicates significant downregulation in aUC, active UC but not rUC, remission UC, whereas in CD, a trend toward reduction is seen in remission (rCD) rather than active disease (aCD). **(b)** Relative transcriptional expression of *VSTM1* (the gene encoding SIRL-1) measured by RT-qPCR (2^-△△CT^). Violin plots display significant suppression of mRNA levels in the total IBD cohort relative to controls (left), driven primarily by the CD subgroup (middle). Stratification by clinical status (right) demonstrates constitutive downregulation in CD across both active (aCD) and remission (rCD) phases, while UC patients show no significant transcriptional deviation from controls. p-values are indicated above the respective comparisons.

Transcriptional data mirrored protein findings, VSTM1 mRNA was suppressed in the total IBD cohort (*p* = 0.0009). This was largely driven by the CD group (*p* = 0.0013). Stratification revealed that while UC patients did not differ significantly from controls, the CD subgroup exhibited sustained transcriptional downregulation in both active (*p* = 0.0398) and remission (*p* = 0.0364) phases. This suggests transcriptional impairment of *VSTM1* is constitutive in CD (**Figure 1**).

### 3.3 Serum IL-8 elevation and transcriptional dysregulation

Serum IL-8 was upregulated in the total IBD cohort (*p* = 0.0025; median 74.71 vs. 17.95 pg/ml). The UC subgroup showed a significant increase (*p* = 0.0041), particularly in active disease (*p* = 0.0534). The CD subgroup did not differ significantly from baseline overall, though active CD showed a trend toward elevation (*p* = 0.0864).

**Figure 2:**
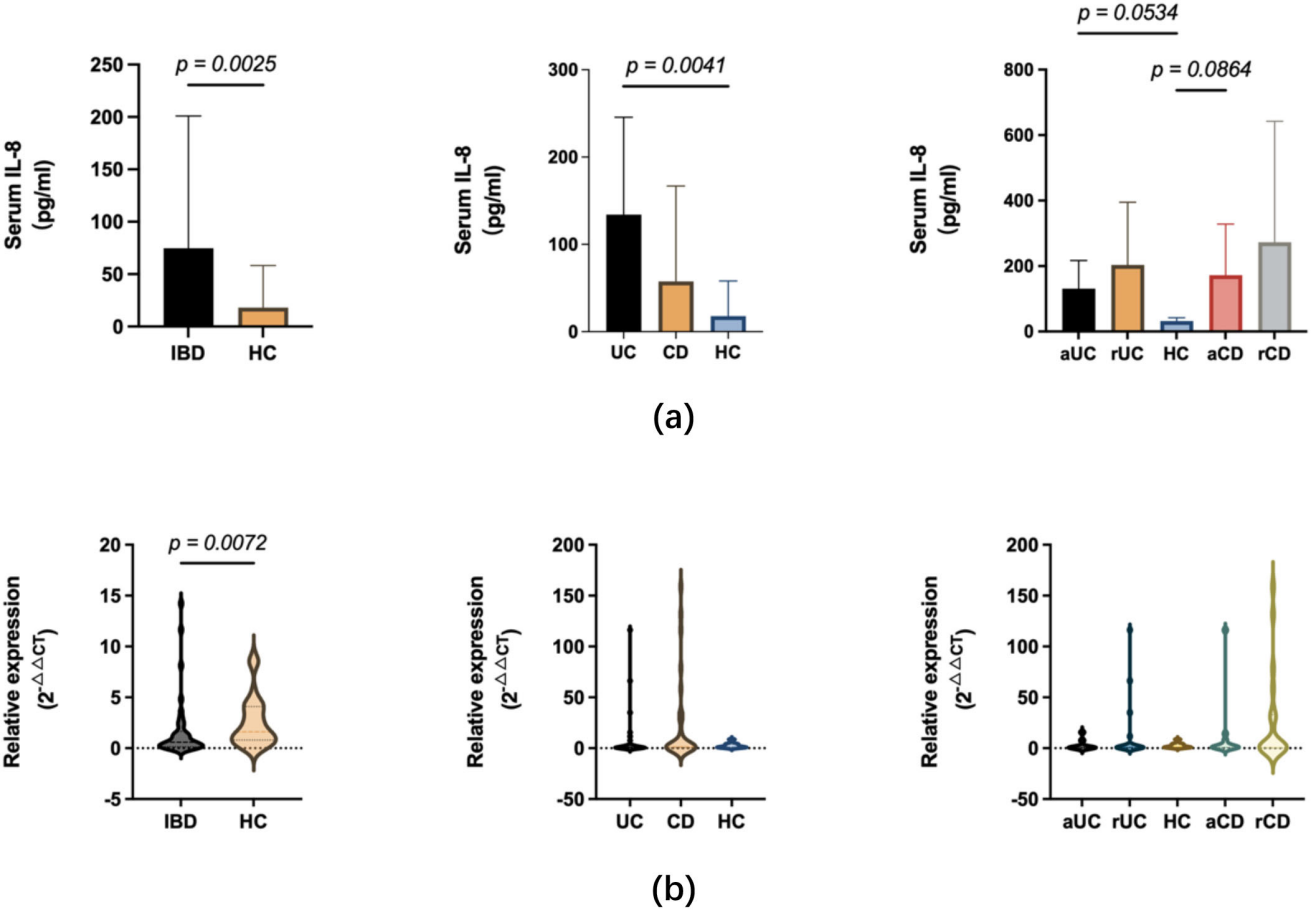
Divergent patterns of serum IL-8 protein and IL8 mRNA expression. **(a)** Serum IL-8 concentrations (pg/ml). Bar graphs depict significantly elevated levels in the total IBD cohort compared to HC, healthy controls (left), driven largely by the UC subgroup (middle). Analysis by clinical activity (right) indicates trends toward increased serum IL-8 in aUC, active UC and aCD, active CD, though these did not reach statistical significance compared to baseline. **(b)** Relative IL8 mRNA expression in peripheral blood (2^-△△CT^). In contrast to serum protein findings, violin plots show significant transcriptional downregulation in the IBD cohort relative to controls (left). Stratification into UC and CD subgroups (middle) and by disease activity phases (right) reveals no significant differences, suggesting the transcriptional repression observed in the IBD data is not preserved at the subgroup level. p-values are displayed above relevant comparisons.

In contrast to serum protein elevation, peripheral blood IL8 mRNA was downregulated in the aggregate IBD cohort (*p* = 0.0072). However, subgroup analysis showed no significant.

differences for UC (*p* = 0.9571) or CD (*p* > 0.9999) relative to controls, regardless of disease activity. This suggests peripheral IL-8 transcriptional repression is subtle and not robust at the subgroup level (**Figure 2**).

### 3.4 Inverse correlation between SIRL-1 and IL-8

**Figure 3:**
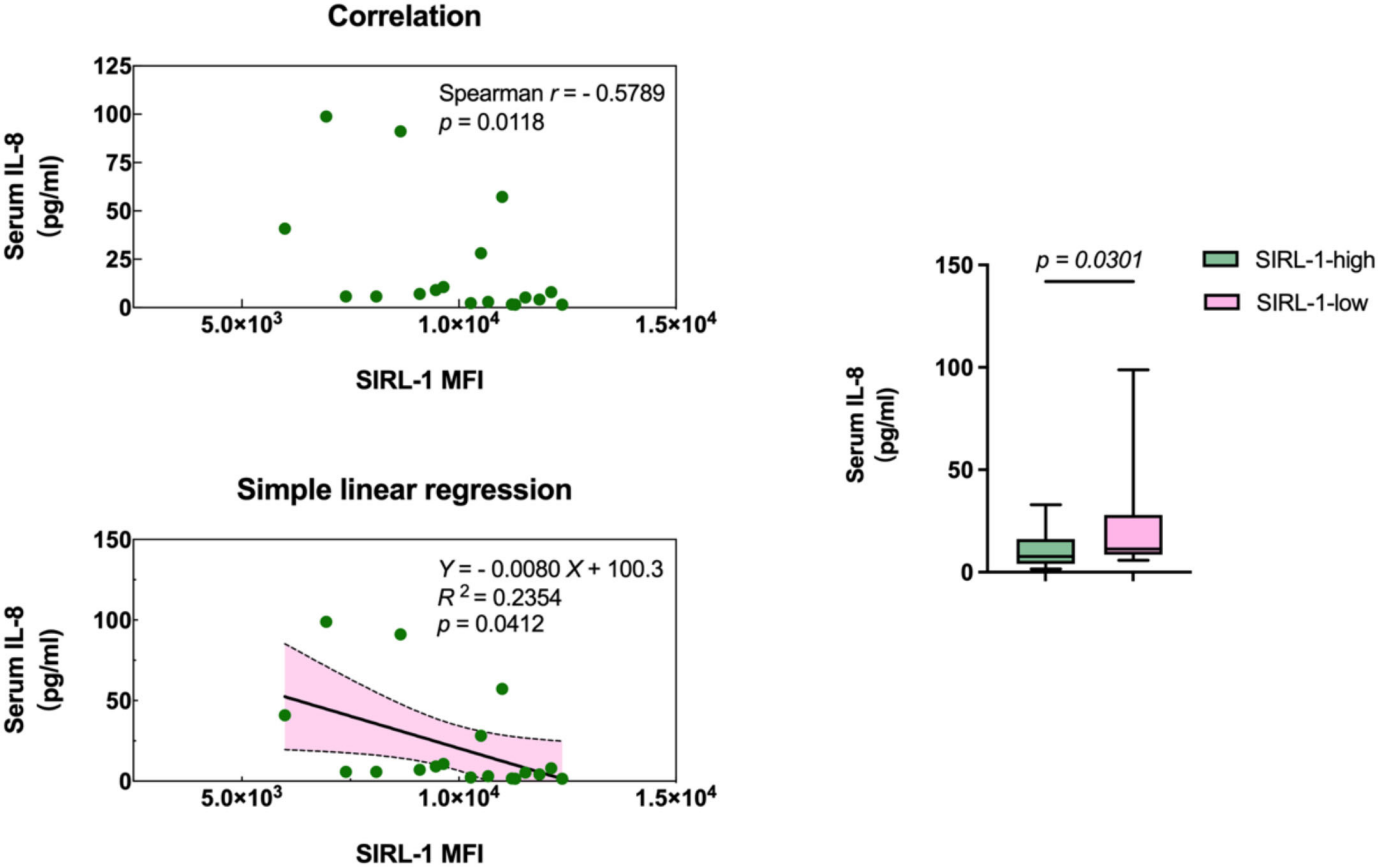
Inverse association between neutrophil SIRL-1 expression and serum IL-8 levels. Spearman correlation analysis (top left) demonstrates a significant negative relationship between SIRL-1 MFI and serum IL-8 concentration (r = -0.5789, *p* = 0.0118). Simple linear regression (bottom left) confirms this inverse dependency (R^2^ = 0.2354, *p* = 0.0412), with the shaded area representing the 95% confidence interval. Box plot comparison (right) of serum IL-8 levels stratified by SIRL-1 expression (median split) shows significantly higher IL-8 concentrations in the SIRL-1-low phenotype compared to the SIRL-1-high group (*p* = 0.0301).

Spearman analysis revealed a negative correlation between neutrophil SIRL-1 MFI and serum IL-8 (r = -0.5789, *p* = 0.0118). Linear regression confirmed this relationship (*p* = 0.0412, R^2^ = 0.2354), indicating SIRL-1 expression accounts for approximately 23.5% of IL-8 variance. Stratification into SIRL-1-high and SIRL-1-low groups (median split) showed significantly higher IL-8 in the low-expression phenotype (*p* = 0.0301), supporting the hypothesis that SIRL-1 deficiency permits IL-8 overexpression (**Figure 3**).

### 3.5 Clinical associations of IL-8 and SIRL-1 with disease activity

**Figure 4:**
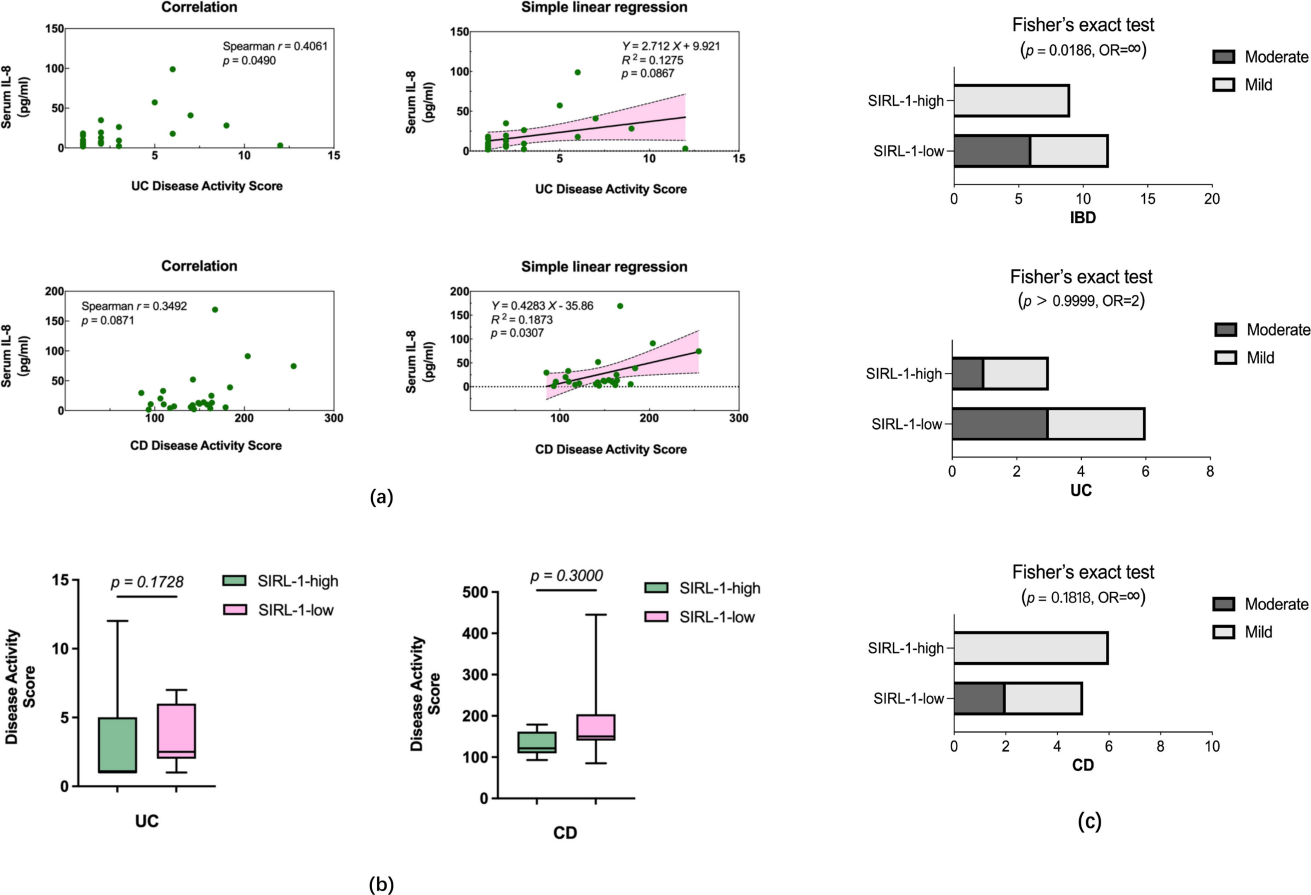
Association of serum IL-8 and SIRL-1 expression with clinical disease activity. **(a)** Correlation between serum IL-8 levels and disease severity. Top panels: Spearman correlation (left; r = 0.4061, *p* = 0.0490) and linear regression (right; R^2^ = 0.1275, *p* = 0.0867) of IL-8 versus UC Disease Activity Score. Bottom panels: Spearman correlation (left; r = 0.3492, *p* = 0.0871) and linear regression (right; R^2^ = 0.1873, *p* = 0.0307) of IL-8 versus CD Disease Activity Score. **(b)** Box plots comparing disease activity scores between SIRL-1-high and SIRL-1-low expression groups. No significant differences were observed in either UC (*p* = 0.1728) or CD (*p* = 0.3000). **(c)** Fisher’s exact test analysis of SIRL-1 expression status (high vs. low) stratified by clinical phenotype (mild vs. moderate). Bar charts display the distribution for the total IBD cohort (top), UC subgroup (middle), and CD subgroup (bottom). No significant associations were found between SIRL-1 status and categorical disease severity in the UC or CD cohort (*p* > 0.05 for all comparisons).

Serum IL-8 correlated with disease severity in UC (Spearman r = 0.4061, *p* = 0.0490). In CD, linear regression indicated a positive relationship between IL-8 and CDAI (*p* = 0.0307). In contrast, SIRL-1 MFI did not correlate linearly with activity scores in either UC (*p* = 0.1728) or CD (*p* = 0.3000) when comparing high vs. low expression groups. Fisher’s exact test showed no association between SIRL-1 status and mild/moderate disease phenotypes in the UC or CD subgroup (**Figure 4**).

### 3.6 Diagnostic efficacy for IBD and disease subtypes

ROC analysis evaluated SIRL-1 MFI, serum IL-8, and the SIRL-1/IL-8 ratio. In the total IBD cohort, the SIRL-1/IL-8 ratio (AUC = 0.82, 95% CI: 0.71-0.93, *p* < 0.0001) outperformed SIRL-1 (AUC = 0.69, 95% CI: 0.57-0.81, *p* = 0.0032) and IL-8 (AUC = 0.70, 95% CI: 0.58-0.83, *p* = 0.0028) alone. At an optimal cutoff value of 2009, the ratio achieved the sensitivity of 89.47% and specificity to 77.36%.

**Figure 5:**
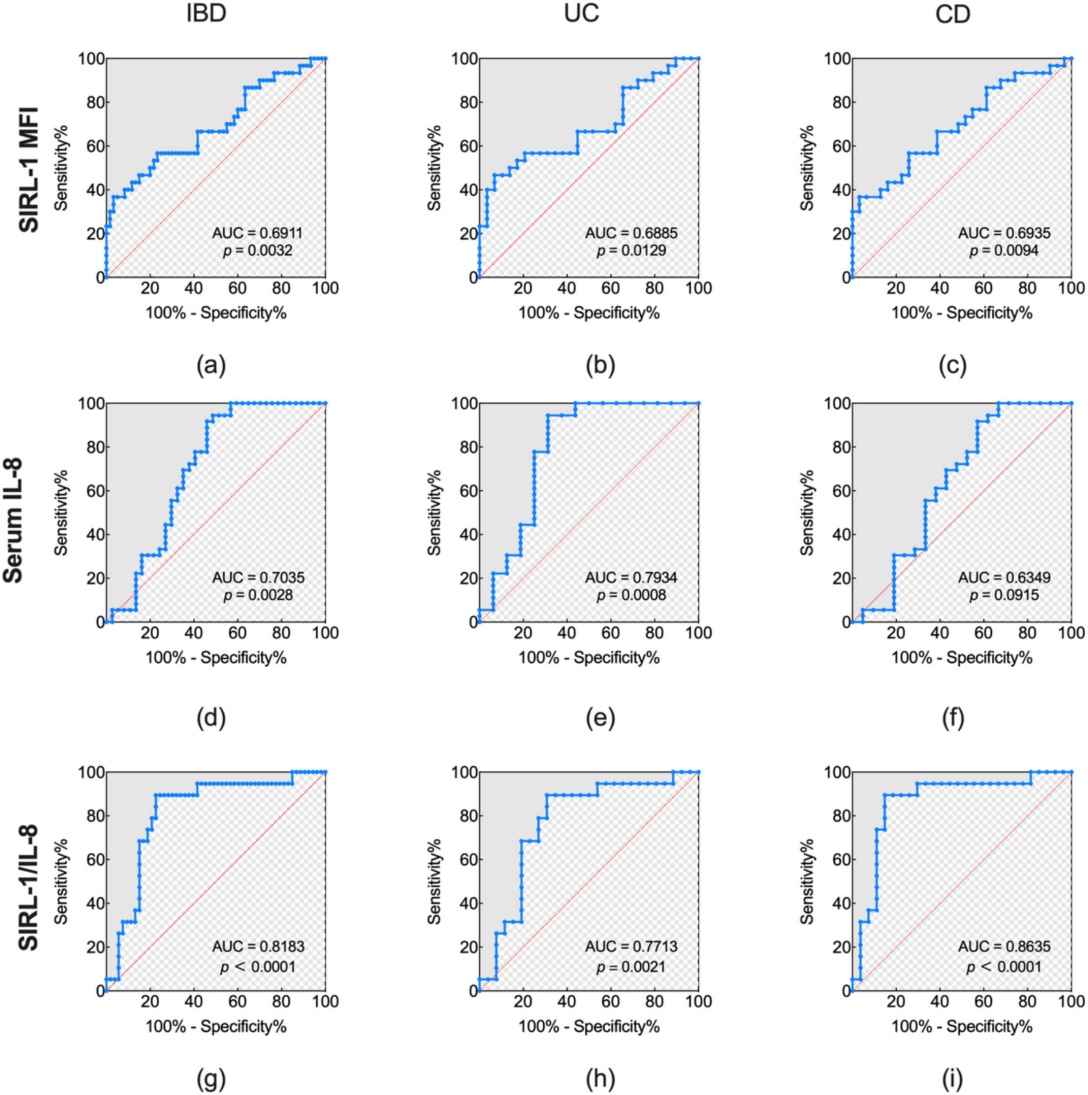
ROC, receiver operating characteristic curve analysis of SIRL-1, IL-8, and the SIRL-1/IL-8 ratio for IBD diagnosis. (a–c) SIRL-1 MFI: Diagnostic performance of neutrophil SIRL-1 expression in the total IBD cohort **(a)** (AUC = 0.6911, *p* = 0.0032), UC subgroup **(b)** (AUC = 0.6885, *p* = 0.0129), and CD subgroup **(c)** (AUC = 0.6935, *p* = 0.0094). (d–f) Serum IL-8: Diagnostic efficacy of serum IL-8 levels in the total IBD cohort **(d)** (AUC = 0.7035, *p* = 0.0028), UC subgroup **(e)** (AUC = 0.7934, *p* = 0.0008), and CD subgroup **(f)** (AUC = 0.6349, *p* = 0.0915). Note the lack of statistical significance for IL-8 in distinguishing CD from controls. **(g–i)** SIRL-1/IL-8 Ratio: Diagnostic accuracy of the combined ratio in the total IBD cohort **(g)** (AUC = 0.8183, *p* < 0.0001), UC subgroup **(h)** (AUC = 0.7713, *p* = 0.0021), and CD subgroup **(i)** (AUC = 0.8635, *p* < 0.0001). The ratio demonstrates superior discriminative ability compared to individual markers, particularly in the CD cohort where it achieved the highest observed AUC.

In UC, IL-8 was a strong predictor with 94.44% sensitivity (AUC = 0.79, 95% CI: 0.63-0.96, *p* = 0.0008), comparable to the SIRL-1/IL-8 ratio (AUC = 0.77, 95% CI: 0.63-0.92, *p* = 0.0021). However, the ratio maintained the diagnostic value with a robust specificity (69.23%) while the sensitivity reached 89.47%. For this cohort, SIRL-1 showed modest discrimination (AUC = 0.69, *p* = 0.0129). In contrast, IL-8 performed poorly (AUC = 0.63, *p* = 0.0915) in the CD cohort, whereas the SIRL-1/IL-8 ratio exhibited the highest diagnostic accuracy (AUC = 0.86, 95% CI: 0.75-0.98, *p* < 0.0001), overcoming the limitations of using either inflammation (IL-8) or regulation (SIRL-1) markers in isolation. Moreover, the ratio performed an effective stratification of CD patients with a diagnostic odds ratio (DOR) of 48.87, suggesting a superior potential at both the high sensitivity (89.47%) and specificity (85.19%) (**Figure 5**; **Table 1**).

**Table 1:** Comparison of diagnostic efficacy for IBD and disease subtypes.

Disease	Variable	AUC (95% CI)	Cut-off	Sensitivity (%)	Specificity (%)	Youden Index	L.R.+	L.R.-	DOR	*P*-value
IBD	SIRL-1 **(a)**	0.69 (0.57-0.81)	12439	36.67	96.67	0.33	11.01	0.66	16.81	0.0032
	IL-8 **(d)**	0.70 (0.58-0.83)	73.81	94.44	51.35	0.46	1.94	0.11	17.93	0.0028
	SIRL-1/IL-8 **(g)**	0.82 (0.71-0.93)	2009	89.47	77.36	0.67	3.95	0.14	29.03	<0.0001
UC	SIRL-1 **(b)**	0.69 (0.55-0.83)	11922	46.67	93.10	0.40	6.76	0.57	11.81	0.0129
	IL-8 **(e)**	0.79 (0.63-0.96)	73.81	94.44	68.75	0.63	3.02	0.08	37.37	0.0008
	SIRL-1/IL-8 **(h)**	0.77 (0.63-0.92)	1885	89.47	69.23	0.59	2.91	0.15	19.12	0.0021
CD	SIRL-1 **(c)**	0.69 (0.56-0.83)	12439	36.67	96.77	0.33	11.35	0.65	17.35	0.0094
	IL-8 **(f)**	0.63 (0.47-0.80)	70.11	91.67	42.86	0.35	1.60	0.19	8.25	0.0915
	SIRL-1/IL-8 **(i)**	0.86 (0.75-0.98)	2009	89.47	85.19	0.75	6.04	0.12	48.87	<0.0001

L.R.+, Positive Likelihood Ratio; L.R.-, Negative Likelihood Ratio; DOR, Diagnostic Odds Ratio; Bold letters (a-i) refer to the corresponding ROC curve panels in **Figure 5**.

### 3.7 Diagnostic value for monitoring disease activity

Differentiation between active and remission states was phenotype-dependent. In the IBD population, only the SIRL-1/IL-8 ratio yielded significant result (AUC = 0.67, 95% CI: 0.51-0.83, *p* = 0.0347). Its sensitivity and specificity at the optimal cutoff of 967.4 were 61.29% and 77.27%, respectively. Either SIRL-1 (AUC = 0.56, *p* = 0.4580) or IL-8 (AUC = 0.61, *p* = 0.2740) alone had no statistically distinct.

**Figure 6:**
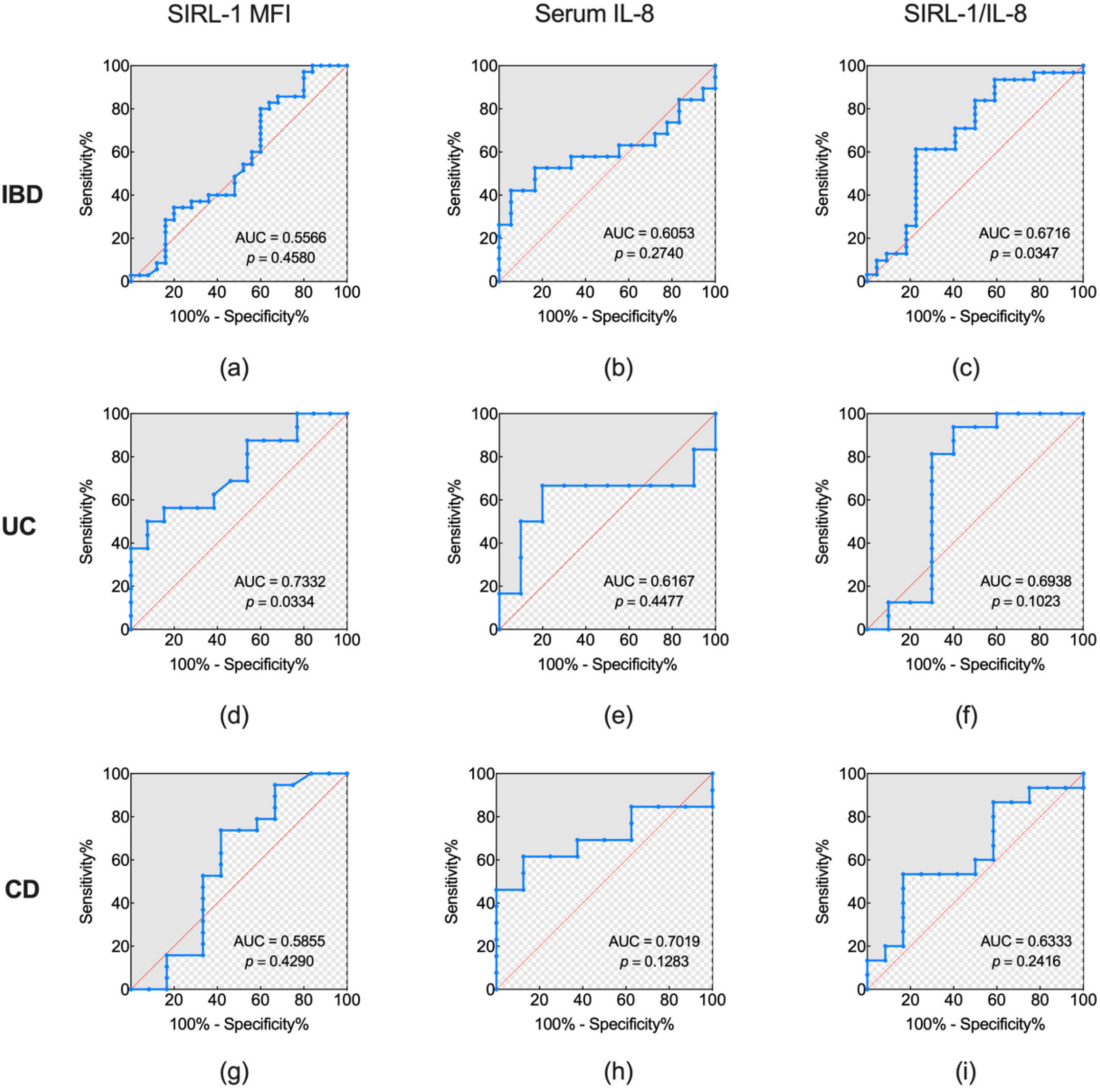
ROC, receiver operating characteristic curve analysis for differentiating active from remission disease states. ROC curves illustrate the diagnostic performance of SIRL-1 MFI, serum IL-8, and the SIRL-1/IL-8 ratio in distinguishing between active and remission phases within different IBD cohorts. SIRL-1 MFI performance is shown for the total IBD cohort **(a)** (AUC = 0.5598, *p* = 0.4580), UC, Ulcerative Colitis subgroup **(d)** (AUC = 0.7332, *p* = 0.0334), and CD, Crohn’s Disease subgroup **(g)** (AUC = 0.5955, *p* = 0.4290), demonstrating significant discriminative power only in the UC subgroup. Serum IL-8 performance is presented for the total IBD cohort **(b)** (AUC = 0.6053, *p* = 0.2740), UC subgroup **(e)** (AUC = 0.6157, *p* = 0.4477), and CD subgroup **(h)** (AUC = 0.7019, *p* = 0.1283), with no significant distinction between active and remission states observed in any cohort. The SIRL-1/IL-8 ratio performance is depicted for the total IBD cohort **(c)** (AUC = 0.6716, *p* = 0.0347), UC subgroup **(f)** (AUC = 0.6938, *p* = 0.1023), and CD subgroup **(i)** (AUC = 0.6333, *p* = 0.2416), achieving statistical significance only in the total IBD cohort for differentiating active from remission disease.

Conversely, in the UC subgroup, the SIRL-1/IL-8 ratio didn’t reach statistical significance (AUC = 0.69, *p* = 0.1023), even though the biomarker exhibited high sensitivity (93.75%). Similarly, IL-8 alone showed poor discriminative power in this section (AUC = 0.62, *p* = 0.4477). SIRL-1 emerged as a sole indicator of active UC with the AUC of 0.73 (95% CI: 0.55-0.92, *p* = 0.0334), the specificity of which reached 92.31%. Meanwhile, no markers effectively distinguished active phase from remission in CD patients: IL-8 (AUC = 0.70, *p* = 0.1283); SIRL-1 (AUC = 0.59, *p* = 0.4290); the SIRL-1/IL-8 ratio (AUC = 0.63, *p* = 0.2416) (**Figure 6**; **Table 2**).

**Table 2:** Comparison of diagnostic value for monitoring disease activity.

Activity	Variable	AUC (95% CI)	Cut-off	Sensitivity (%)	Specificity (%)	Youden Index	L.R.+	L.R.-	DOR	*P*-value
IBD	SIRL-1 **(a)**	0.56 (0.40-0.71)	8716	80.00	40.00	0.20	1.33	0.50	2.67	0.4580
	IL-8 **(b)**	0.61 (0.41-0.80)	10.83	42.11	94.44	0.37	7.57	0.61	12.36	0.2740
	SIRL-1/IL-8 **(c)**	0.67 (0.51-0.83)	967.4	61.29	77.27	0.39	2.70	0.50	5.38	0.0347
UC	SIRL-1 **(d)**	0.73 (0.55-0.92)	11096	50.00	92.31	0.42	6.50	0.54	12.00	0.0334
	IL-8 **(e)**	0.62 (0.27-0.96)	168.9	66.67	80.00	0.47	3.33	0.42	8.00	0.4477
	SIRL-1/IL-8 **(f)**	0.69 (0.44-0.95)	515.6	93.75	60.00	0.54	2.34	0.10	22.50	0.1023
CD	SIRL-1 **(g)**	0.59 (0.35-0.82)	10486	73.68	58.33	0.32	1.77	0.45	3.92	0.4290
	IL-8 **(h)**	0.70 (0.48-0.93)	47.85	61.54	87.5	0.49	4.92	0.44	11.20	0.1283
	SIRL-1/IL-8 **(i)**	0.63 (0.42-0.85)	967.4	53.33	83.33	0.37	3.20	0.56	5.71	0.2416

L.R.+: Positive Likelihood Ratio; L.R.-: Negative Likelihood Ratio; DOR: Diagnostic Odds Ratio; Bold letters (a-i) refer to the corresponding ROC curve panels in **Figure 6**.

## 4 Discussion

This study identifies systemic downregulation of the inhibitory receptor SIRL-1 as a distinct feature of IBD, exhibiting a constitutive defect pattern in Crohn’s Disease and a state-dependent reduction in Ulcerative Colitis. While previous research established neutrophil recruitment and IL-8 (CXCL8) overproduction as drivers of IBD pathogenesis (25, 26), our data suggest this inflammatory state is compounded by a failure of intrinsic inhibitory checkpoints. SIRL-1 (*VSTM1*) dampens Fc receptor-induced oxidative burst and NETosis in granulocytes (11, 16). The reciprocal relationship where SIRL-1 deficiency correlates with unchecked IL-8 expression suggests that defects in this ITIM-bearing receptor lower the neutrophil activation threshold, similar to observations regarding FcγRIIb in other autoimmune conditions (27). Furthermore, the SIRL-1/IL-8 ratio demonstrates high diagnostic potential, addressing the clinical need for biomarkers that capture complex immune dysregulation, particularly in Crohn’s Disease where simple cytokine elevations are variable (28, 29).

The inverse correlation between neutrophil SIRL-1 and serum IL-8 supports the function of SIRL-1 as a physiological restraint on granulocyte activation. Both SIRL-1 and IL-8 are individually important in the complex disease biology of IBD, with SIRL-1 acting as a crucial immune checkpoint and IL-8 being a potent pro-inflammatory mediator. However, the moderate correlation coefficient observed between these two single parameters likely reflects the non-linear dynamics of immune regulation, where SIRL-1 acts as a threshold “brake” that recruits SHP-1 and SHP-2 phosphatases to dephosphorylate signaling molecules involved in oxidative burst and cytokine secretion (11, 30–33). This mechanism parallels other myeloid checkpoints, such as LAIR-1, which suppress pathogenic cytokine loops in chronic inflammation (34). Systemic reduction of SIRL-1 implies a lowered activation threshold, rendering neutrophils hypersensitive to microbial ligands or damage-associated molecular patterns (DAMPs) (35). This disinhibition likely facilitates the exaggerated IL-8 response characteristic of active mucosal inflammation (36, 37). The superior diagnostic accuracy of the SIRL-1/IL-8 ratio supports its biological relevance, capturing the functional imbalance between immune inhibition and activation that more comprehensively characterizes IBD pathology than either marker alone. The robustness of this axis also reinforces the utility of assessing the regulation-to-inflammation ratio over inflammatory burden alone (38).

Our data highlight phenotypic divergence between UC and CD, echoing the molecular heterogeneity of these subtypes (39). Specifically, our results demonstrate that VSTM1 mRNA is significantly downregulated in the total IBD cohort, generally mirroring the reduction in SIRL-1 protein expression. However, subgroup analysis revealed distinct regulatory patterns. In UC, SIRL-1 suppression appeared state-dependent, tracking with disease activity and normalizing in remission, suggesting a secondary response to the acute inflammatory milieu. The modulatory mechanism was observed similarly to other myeloid receptors like TREM-1 in active colitis (40). In contrast, the CD cohort exhibited constitutive transcriptional suppression of *VSTM1* regardless of clinical status (persisting in remission). Interestingly, while mRNA remained suppressed, protein levels in CD remission showed a trend toward normalization, creating a partial discordance. This suggests that in addition to the constitutive transcriptional repression seen in CD, SIRL-1 surface expression is likely fine-tuned by post-transcriptional mechanisms or proteolytic shedding, which may vary with the resolution of inflammation. This persistence of transcriptional defect aligns with GWAS data identifying stable epigenetic alterations in myeloid loci distinguishing CD from UC (41, 42). We postulate that in CD, SIRL-1 deficiency may represent an inherent susceptibility factor or “molecular scar,” leaving patients vulnerable to relapse despite symptomatic control. The phenomenon was previously observed only in other innate immune defects such as ATG16L1 dysfunction in CD (43).

Clinically, these insights improve diagnostic utility. Standard biomarkers like CRP and fecal calprotectin exhibit variable specificity and may not capture transmural healing timely, particularly in small-bowel Crohn’s Disease (44, 45). In our cohort, the SIRL-1/IL-8 ratio achieved an AUC of 0.86 for CD diagnosis, significantly outperforming serum IL-8 alone. This is particularly notable given that serum IL-8 levels were predominantly elevated in UC rather than CD. The high diagnostic accuracy of the ratio in CD indicates that the pathogenic driver may not be the absolute cytokine quantity, but rather the failure of the inhibitory checkpoint (SIRL-1) to counterbalance even basal or distinct inflammatory signals. This enhancement likely stems from normalizing the inflammatory signal against intrinsic regulatory capacity, amplifying the signal-to-noise ratio that is increasingly advocated in precision medicine for autoimmune disorders (46). While a direct statistical comparison with a comprehensive panel of conventional markers within our cohort was not uniformly feasible for robust analysis in this retrospective study, the diagnostic performance of the SIRL-1/IL-8 ratio compares favorably with established literature benchmarks. For instance, a systematic review and meta-analysis by Mosli et al. (44) reported that for detecting endoscopic activity, fecal calprotectin showed high sensitivity (0.88) and moderate specificity (0.73), yielding a Youden index of 0.61 and a DOR of 19.83. Similarly, stool lactoferrin presented comparable overall performance with a sensitivity of 0.82, specificity of 0.79, a Youden index of 0.61 and a DOR of 17.14. In contrast, CRP demonstrated high specificity (0.92) but low sensitivity (0.49), resulting in a lower Youden index (0.41) and DOR (11.05). Beyond these meta-analytical averages, individual studies often reveal significant variability and lower diagnostic efficiency. Specifically for Crohn’s Disease, several studies have consistently highlighted CRP’s limited diagnostic utility, with reported sensitivities often ranging from 48% to 68% and specificities from 58% to 91%, translating to notably lower Youden indices (e.g., 0.26 to 0.39) and DORs (e.g., 2.93 to 9.33) (47–49). While fecal calprotectin and lactoferrin can achieve higher sensitivity and specificity in some specific cohorts or with optimized cutoffs (48), their performance remains variable across different studies (44). Moreover, ESR, a widely used traditional marker, is less reliable for monitoring IBD activity as its levels do not change quickly with disease activity, impacting its utility (28). Regarding serum cytokines, Korolkova et al. (50) found that certain individual cytokines, such as IL-8, were significantly elevated in both UC and Crohn’s colitis patients compared to controls, and IL-6 was elevated in Crohn’s colitis. However, their study explicitly concluded that no single cytokine alone was sufficient to differentiate between UC and Crohn’s colitis, requiring a generalized linear model combining multiple cytokines to achieve a high diagnostic AUC for subtype differentiation. This finding underscores that while individual cytokine levels reflect inflammation, they often lack the specificity or comprehensive information needed for nuanced diagnosis or subtype differentiation. In sharp contrast, our SIRL-1/IL-8 ratio achieved a sensitivity of 89.47% and specificity of 85.19% for distinguishing CD from healthy controls, culminating in a Youden index of 0.75 and a remarkable DOR of 48.87. This suggests that the ratio offers superior diagnostic precision by capturing the functional immune imbalance. This metric may identify the defective immune regulation in distinct patients, contributing to exploring therapeutic strategies targeting inhibitory receptor pathways such like current approaches in rheumatoid arthritis and lupus (51–54).

There are several limitations in the retrospective study. First, it established correlation rather than causality; mechanistic experiments using *VSTM1* knockout or overexpression models are required to confirm the suppression of IL-8 by SIRL-1. Second, peripheral blood profiling was used to serve as a surrogate for intestinal immunity in this work; tissue-resident neutrophils obtained via biopsy would be valuable to validate the mucosal pathophysiology. Third, a specific viability dye was not employed in the flow cytometry analysis due to the retrospective design. However, all samples were processed immediately as fresh whole blood to ensure high cell viability, and strict morphological gating was applied to exclude debris. Given that dead cells typically increase non-specific background signals, the significant downregulation of SIRL-1 observed is unlikely to be an artifact of cell viability. Furthermore, it warrants molecular investigation on post-transcriptional regulatory mechanisms of the discordance between transcript and protein levels in subgroups.

Generally, this study characterizes SIRL-1 deficiency as a permissive factor for the IL-8 inflammatory axis in IBD. The dysregulation of this myeloid checkpoint might promote the chronic inflammation, particularly in Crohn’s Disease where the defect exists persistently in remission. The SIRL-1/IL-8 ratio represents a promising biomarker capturing the interplay between immune regulation and activation systemically, and provides a diagnostic and stratificational tool for patients with IBD.

## Data Availability

The original contributions presented in the study are included in the article/**Supplementary Material**. Further inquiries can be directed to the corresponding authors.

## References

[1] Hisamatsu T Kanai T Mikami Y Yoneno K Matsuoka K Hibi T . Immune aspects of the pathogenesis of inflammatory bowel disease. Pharmacol Ther. (2013) 137:283–97. doi: 10.1016/j.pharmthera.2012.10.008. PMID: 23103332

[2] Xu XR Liu CQ Feng BS Liu ZJ . Dysregulation of mucosal immune response in pathogenesis of inflammatory bowel disease. World J Gastroenterol. (2014) 20:3255–64. doi: 10.3748/wjg.v20.i12.3255. PMID: 24695798 PMC3964397

[3] Fonseca-Camarillo G Yamamoto-Furusho JK . Immunoregulatory pathways involved in inflammatory bowel disease. Inflammation Bowel Dis. (2015) 21:2188–93. doi: 10.1097/MIB.0000000000000477. PMID: 26111210

[4] Hu Y Sun S Li H . Identification and functional exploration of ferroptosis and immune related long non-coding RNA in inflammatory bowel disease (2022). Available online at: https://ww.researchsquare.com/article/rs-1857506/v1 (Accessed July 25, 2022).

[5] Ortega-Zapero M Gomez-Bris R Pascual-Laguna I Saez A Gonzalez-Granado JM . Neutrophils and NETs in pathophysiology and treatment of inflammatory bowel disease. Int J Mol Sci. (2025) 26:7098. doi: 10.3390/ijms26157098. PMID: 40806230 PMC12346662

[6] Zhou GX Liu ZJ . Potential roles of neutrophils in regulating intestinal mucosal inflammation of inflammatory bowel disease. J Dig Dis. (2017) 18:495–503. doi: 10.1111/1751-2980.12540. PMID: 28857501

[7] Mitsuyama K Toyonaga A Sasaki E Watanabe K Tateishi H Nishiyama T . IL-8 as an important chemoattractant for neutrophils in ulcerative colitis and Crohn's disease. Clin Exp Immunol. (1994) 96:432–6. doi: 10.1111/j.1365-2249.1994.tb06047.x. PMID: 8004812 PMC1534558

[8] Reimund JM Duclos B Dumont S Muller C Baumann R Poindron P . Interleukin-8 is an important inflammatory mediator in hemorrhagic rectocolitis and Crohn disease. Gastroenterol Clin Biol. (1997) 21:131–7. 9161479

[9] Ina K Kusugami K Yamaguchi T Imada A Hosokawa T Ohsuga M . Mucosal interleukin-8 is involved in neutrophil migration and binding to extracellular matrix in inflammatory bowel disease. Am J Gastroenterol. (1997) 92:1342–6. 9260803

[10] Cominelli F Pizarro TT . Interleukin-1 and interleukin-1 receptor antagonist in inflammatory bowel disease. Aliment Pharmacol Ther. (1996) 10:49–53. doi: 10.1046/j.1365-2036.1996.22164020.x. PMID: 8899101

[11] Steevels TA Lebbink RJ Westerlaken GH Coffer PJ Meyaard L . Signal inhibitory receptor on leukocytes-1 is a novel functional inhibitory immune receptor expressed on human phagocytes. J Immunol. (2010) 184:4741–8. doi: 10.4049/jimmunol.0902039. PMID: 20375307

[12] Rumpret M von Richthofen HJ van der Linden M Westerlaken GHA Talavera Ormeno C van Strijp JAG . Signal inhibitory receptor on leukocytes-1 recognizes bacterial and endogenous amphipathic alpha-helical peptides. FASEB J. (2021) 35:e21875. doi: 10.1096/fj.202100812R. PMID: 34533845 PMC12316092

[13] Takagane K Umakoshi M Itoh G Kuriyama S Goto A Tanaka M . SKAP2 suppresses inflammation-mediated tumorigenesis by regulating SHP-1 and SHP-2. Oncogene. (2022) 41:1087–99. doi: 10.1038/s41388-021-02153-1. PMID: 35034964

[14] Steevels TA van Avondt K Westerlaken GH Stalpers F Walk J Bont L . Signal inhibitory receptor on leukocytes-1 (SIRL-1) negatively regulates the oxidative burst in human phagocytes. Eur J Immunol. (2013) 43:1297–308. doi: 10.1002/eji.201242916. PMID: 23436183

[15] Van Avondt K Fritsch-Stork R Derksen RH Meyaard L . Ligation of signal inhibitory receptor on leukocytes-1 suppresses the release of neutrophil extracellular traps in systemic lupus erythematosus. PloS One. (2013) 8:e78459. doi: 10.1371/journal.pone.0078459. PMID: 24205237 PMC3799702

[16] Van Avondt K van der Linden M Naccache PH Egan DA Meyaard L . Signal inhibitory receptor on leukocytes-1 limits the formation of neutrophil extracellular traps, but preserves intracellular bacterial killing. J Immunol. (2016) 196:3686–94. doi: 10.4049/jimmunol.1501650. PMID: 27016607

[17] Koops M Meyaard L . VSTM1/SIRL-1: An inhibitory pattern recognition receptor regulating myeloid cells. Eur J Immunol. (2025) 55:e202451465. doi: 10.1002/eji.202451465. PMID: 39989259 PMC11848704

[18] Kumar D Puan KJ Andiappan AK Lee B Westerlaken GH Haase D . A functional SNP associated with atopic dermatitis controls cell type-specific methylation of the VSTM1 gene locus. Genome Med. (2017) 9:18. doi: 10.1186/s13073-017-0404-6. PMID: 28219444 PMC5319034

[19] Xv Z Xv X Chen N Yuan J Li J Wang L . Soluble signal inhibitory receptor on leukocytes-1 reflects disease activity and assists diagnosis of patients with rheumatoid arthritis. Clin Chim Acta. (2024) 556:117808. doi: 10.1016/j.cca.2024.117808. PMID: 38309555

[20] von Richthofen HJ Westerlaken GHA Gollnast D Besteman S Delemarre EM Rodenburg K . Soluble signal inhibitory receptor on leukocytes-1 is released from activated neutrophils by proteinase 3 cleavage. J Immunol. (2023) 210:389–97. doi: 10.4049/jimmunol.2200169. PMID: 36637221 PMC9915861

[21] Guo X Zhang Y Wang P Li T Fu W Mo X . VSTM1-v2, a novel soluble glycoprotein, promotes the differentiation and activation of Th17 cells. Cell Immunol. (2012) 278:136–42. doi: 10.1016/j.cellimm.2012.07.009. PMID: 22960280

[22] von Richthofen HJ Hafkamp FMJ van Haperen A de Jong EC Meyaard L . VSTM1-v2 does not drive human Th17 cell differentiation: A replication study. PloS One. (2023) 18:e0284404. doi: 10.1371/journal.pone.0284404. PMID: 37053248 PMC10101491

[23] Rumpret M von Richthofen HJ van der Linden M Westerlaken GHA Talavera Ormeno C Low TY . Recognition of S100 proteins by signal inhibitory receptor on leukocytes-1 negatively regulates human neutrophils. Eur J Immunol. (2021) 51:2210–7. doi: 10.1002/eji.202149278. PMID: 34145909 PMC8457157

[24] von Richthofen HJ Gollnast D van Capel TMM Giovannone B Westerlaken GHA Lutter L . Signal inhibitory receptor on leukocytes-1 is highly expressed on lung monocytes, but absent on mononuclear phagocytes in skin and colon. Cell Immunol. (2020) 357:104199. doi: 10.1016/j.cellimm.2020.104199. PMID: 32942189

[25] Russo RC Garcia CC Teixeira MM Amaral FA . The CXCL8/IL-8 chemokine family and its receptors in inflammatory diseases. Expert Rev Clin Immunol. (2014) 10:593–619. doi: 10.1586/1744666X.2014.894886. PMID: 24678812

[26] Pearl DS Shah K Whittaker MA Nitch-Smith H Brown JF Shute JK . Cytokine mucosal expression in ulcerative colitis, the relationship between cytokine release and disease activity. J Crohns Colitis. (2013) 7:481–9. doi: 10.1016/j.crohns.2012.07.022. PMID: 22974822

[27] Smith KG Clatworthy MR . FcgammaRIIB in autoimmunity and infection: evolutionary and therapeutic implications. Nat Rev Immunol. (2010) 10:328–43. doi: 10.1038/nri2762. PMID: 20414206 PMC4148599

[28] Lewis JD . The utility of biomarkers in the diagnosis and therapy of inflammatory bowel disease. Gastroenterology. (2011) 140:1817–26. doi: 10.1053/j.gastro.2010.11.058. PMID: 21530748 PMC3749298

[29] Gaya DR Russell RK Nimmo ER Satsangi J . New genes in inflammatory bowel disease: lessons for complex diseases? Lancet. (2006) 367:1271–84. doi: 10.1016/S0140-6736(06)68345-1. PMID: 16631883

[30] Christofides A Katopodi XL Cao C Karagkouni D Aliazis K Yenyuwadee S . SHP-2 and PD-1-SHP-2 signaling regulate myeloid cell differentiation and antitumor responses. Nat Immunol. (2023) 24:55–68. doi: 10.1038/s41590-022-01385-x. PMID: 36581713 PMC9810534

[31] Lee JH Chiang SY Nam D Chung WS Lee J Na YS . Capillarisin inhibits constitutive and inducible STAT3 activation through induction of SHP-1 and SHP-2 tyrosine phosphatases. Cancer Lett. (2014) 345:140–8. doi: 10.1016/j.canlet.2013.12.008. PMID: 24333736

[32] Speranza L Pesce M Patruno A Franceschelli S Lutiis MA Grilli A . Astaxanthin treatment reduced oxidative induced pro-inflammatory cytokines secretion in U937: SHP-1 as a novel biological target. Mar Drugs. (2012) 10:890–9. doi: 10.3390/md10040890. PMID: 22690149 PMC3366681

[33] Gruber RC LaRocca D Minchenberg SB Christophi GP Hudson CA Ray AK . The control of reactive oxygen species production by SHP-1 in oligodendrocytes. Glia. (2015) 63:1753–61. doi: 10.1002/glia.22842. PMID: 25919645 PMC4534322

[34] Meyaard L . The inhibitory collagen receptor LAIR-1 (CD305). J Leukoc Biol. (2008) 83:799–803. doi: 10.1189/jlb.0907609. PMID: 18063695

[35] El-Benna J Hurtado-Nedelec M Marzaioli V Marie JC Gougerot-Pocidalo MA Dang PM . Priming of the neutrophil respiratory burst: role in host defense and inflammation. Immunol Rev. (2016) 273:180–93. doi: 10.1111/imr.12447. PMID: 27558335

[36] Bennike TB Carlsen TG Ellingsen T Bonderup OK Glerup H Bogsted M . Neutrophil extracellular traps in ulcerative colitis: a proteome analysis of intestinal biopsies. Inflammation Bowel Dis. (2015) 21:2052–67. doi: 10.1097/MIB.0000000000000460. PMID: 25993694 PMC4603666

[37] Friedrich M Pohin M Powrie F . Cytokine networks in the pathophysiology of inflammatory bowel disease. Immunity. (2019) 50:992–1006. doi: 10.1016/j.immuni.2019.03.017. PMID: 30995511

[38] Sands BE . Biomarkers of inflammation in inflammatory bowel disease. Gastroenterology. (2015) 149:1275–85. doi: 10.1053/j.gastro.2015.07.003. PMID: 26166315

[39] Cleynen I Boucher G Jostins L Schumm LP Zeissig S Ahmad T . Inherited determinants of Crohn's disease and ulcerative colitis phenotypes: a genetic association study. Lancet. (2016) 387:156–67. doi: 10.1016/S0140-6736(15)00465-1. PMID: 26490195 PMC4714968

[40] Schenk M Bouchon A Seibold F Mueller C . TREM-1-expressing intestinal macrophages crucially amplify chronic inflammation in experimental colitis and inflammatory bowel diseases. J Clin Invest. (2007) 117:3097–106. doi: 10.1172/JCI30602. PMID: 17853946 PMC1974863

[41] Jostins L Ripke S Weersma RK Duerr RH McGovern DP Hui KY . Host-microbe interactions have shaped the genetic architecture of inflammatory bowel disease. Nature. (2012) 491:119–24. doi: 10.1038/nature11582. PMID: 23128233 PMC3491803

[42] Ventham NT Kennedy NA Adams AT Kalla R Heath S O'Leary KR . Integrative epigenome-wide analysis demonstrates that DNA methylation may mediate genetic risk in inflammatory bowel disease. Nat Commun. (2016) 7:13507. doi: 10.1038/ncomms13507. PMID: 27886173 PMC5133631

[43] Murthy A Li Y Peng I Reichelt M Katakam AK Noubade R . A Crohn's disease variant in Atg16l1 enhances its degradation by caspase 3. Nature. (2014) 506:456–62. doi: 10.1038/nature13044. PMID: 24553140

[44] Mosli MH Zou G Garg SK Feagan SG MacDonald JK Chande N . C-reactive protein, fecal calprotectin, and stool lactoferrin for detection of endoscopic activity in symptomatic inflammatory bowel disease patients: a systematic review and meta-analysis. Am J Gastroenterol. (2015) 110:802–19. doi: 10.1038/ajg.2015.120. PMID: 25964225

[45] Vermeire S Van Assche G Rutgeerts P . C-reactive protein as a marker for inflammatory bowel disease. Inflammation Bowel Dis. (2004) 10:661–5. doi: 10.1097/00054725-200409000-00026. PMID: 15472532

[46] Maecker HT Lindstrom TM Robinson WH Utz PJ Hale M Boyd SD . New tools for classification and monitoring of autoimmune diseases. Nat Rev Rheumatol. (2012) 8:317–28. doi: 10.1038/nrrheum.2012.66. PMID: 22647780 PMC3409841

[47] Solem CA Loftus EV Jr. Tremaine WJ Harmsen WS Zinsmeister AR Sandborn WJ . Correlation of C-reactive protein with clinical, endoscopic, histologic, and radiographic activity in inflammatory bowel disease. Inflamm Bowel Dis. (2005) 11:707–12. doi: 10.1097/01.mib.0000173271.18319.53, PMID: 16043984

[48] Sipponen T Savilahti E Kolho KL Nuutinen H Turunen U Farkkila M . Crohn's disease activity assessed by fecal calprotectin and lactoferrin: correlation with Crohn's disease activity index and endoscopic findings. Inflammation Bowel Dis. (2008) 14:40–6. doi: 10.1002/ibd.20312. PMID: 18022866

[49] Schoepfer AM Beglinger C Straumann A Trummler M Vavricka SR Bruegger LE . Fecal calprotectin correlates more closely with the simple endoscopic score for Crohn's disease (SES-CD) than CRP, blood leukocytes, and the CDAI. Am J Gastroenterol. (2010) 105:162–9. doi: 10.1038/ajg.2009.545. PMID: 19755969

[50] Korolkova OY Myers JN Pellom ST Wang L M'Koma AE . Characterization of serum cytokine profile in predominantly colonic inflammatory bowel disease to delineate ulcerative and Crohn's colitides. Clin Med Insights Gastroenterol. (2015) 8:29–44. doi: 10.4137/CGast.S20612. PMID: 26078592 PMC4459555

[51] Keir ME Butte MJ Freeman GJ Sharpe AH . PD-1 and its ligands in tolerance and immunity. Annu Rev Immunol. (2008) 26:677–704. doi: 10.1146/annurev.immunol.26.021607.090331. PMID: 18173375 PMC10637733

[52] Son M Santiago-Schwarz F Al-Abed Y Diamond B . C1q limits dendritic cell differentiation and activation by engaging LAIR-1. Proc Natl Acad Sci USA. (2012) 109:E3160–7. doi: 10.1073/pnas.1212753109. PMID: 23093673 PMC3503216

[53] Son M Diamond B . C1q-mediated repression of human monocytes is regulated by leukocyte-associated Ig-like receptor 1 (LAIR-1). Mol Med. (2015) 20:559–68. doi: 10.2119/molmed.2014.00185. PMID: 25247291 PMC4365071

[54] Zhang Y Lv K Zhang CM Jin BQ Zhuang R Ding Y . The role of LAIR-1 (CD305) in T cells and monocytes/macrophages in patients with rheumatoid arthritis. Cell Immunol. (2014) 287:46–52. doi: 10.1016/j.cellimm.2013.12.005. PMID: 24380839

